# The five times sit-to-stand test: safety, validity and reliability with critical care survivors’s at ICU discharge

**DOI:** 10.1186/s40945-022-00156-z

**Published:** 2022-12-18

**Authors:** A. Thiago Araújo de Melo, B. Fernando Silva Guimarães, C. José Roberto Lapa e Silva

**Affiliations:** 1 School of Health Sciences – Brazil, Universidade Federal do Rio de Janeiro, Rua das Patativas, 449, Imbuí, Salvador, Bahia Brazil; 2 Physical Therapy Department, Federal University of Rio de Janeiro, Rua prof. Rodolpho Paulo Rocco, 255, oitavo andar, sala 3 - Cidade Universitária da Universidade Federal do Rio de Janeiro, Rio de Janeiro, RJ Brazil; 3 School of Medicine – Federal University of Rio de Janeiro, Rua Ferreira Pontes, 264/103, Andaraí, Rio de Janeiro, RJ Brazil

**Keywords:** ICU, Five Times Sit-to-Stand Test (FTSST), Physical therapy, Rehabilitation

## Abstract

**Background:**

The Five Times Sit-to-Stand Test (FTSST) has been found reliable, safe and valid for measuring healthy adults’ lower limb muscle strength and for determining balance control, fall risk, and exercise capacity among older examinees. We believe that the FTSST has the potential to be a straightforward, low cost and valuable tool for identifying muscle disability and functional status following critical illness. The aim of our study was to establish the applicability, safety, and psychometric qualities of FTSST in patients at Intensive Care Unit (ICU) discharge.

**Methods:**

In our study applicability was determined by assessing the percentage of patients who could perform the test at ICU discharge. Safety was assessed by examining data regarding any exacerbated haemodynamic and respiratory responses or adverse events associated with the test. For assessing FTSST reliability, intraclass correlation coefficients (ICCs), standard error of measurement (SEM) and Bland-Altman plot were used. For assessing concurrent validity handgrip strength, ICU length of stay, duration of invasive ventilation, Simplified Acute Physiology Score 3 (SAPS3) and age variables were used. For investigating predictive validity, correlations between the FTSST and measures of hospital length of stay and functional independence were evaluated.

**Results:**

Only 30% of ICU survivors (*n* = 261 out of 817) were eligible to perform the FTSST and 7% of patients who performed the test (*n* = 10 out of 142) presented adverse events. Both inter (ICC 0.92 CI95% 0.89–0.94) and intra-rater (ICC 0.95 CI95% 0.93–0.96) reliability were excellent and higher scores were associated with lower muscle strength, longer hospital stay and greater functional impairment at hospital discharge in adult survivors of critical diseases.

**Conclusion:**

Our results suggest that the FTSST may be applicable only to high-functioning critical care survivors. In this specifical population, FTSST is a safe, easy to perform, valid and reliable measure that can be applied to fall risk and functional recovery management.

## Key messages

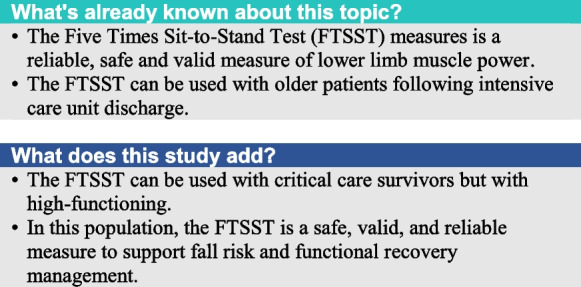



## Background

Sit-to-stand ability requires coordination between the trunk and lower limbs, balance, stability, neurocognitive skills, and muscular strength [1–3]; and it is significantly associated with independence for activities of daily living (ADLs) [4–6]. Some authors have reported that significant functional limitations can occur when the ability to rise from a seat is impaired [7, 8].

The Five Times Sit-to-Stand Test measures the time taken to stand five times from a sitting position as fast as possible [9]. It has been shown to be a reliable, safe and valid measure of lower limb muscle power in critical care survivors [10] and of strength in healthy adults and in persons with various diseases [11]. Other studies reported that the Five Times Sit-to-Stand Test (FTSST) is a valid and reliable measure of balance control and fall risk in older people [12, 13] and of exercise capacity in patients with COPD [14]. Poor performances on FTSST have been associated with an increased risk for recurrent falls [15], slow gait speed [16] and reduced ability to perform ADLs [17]. Past research has produced normative values and predictive and concurrent validity data and test reliability for patients with osteoarthritis [18], stroke [19, 20], Parkinson’s disease [21], and low back pain [22]. Recently, Melo et al. [9] published an evaluation of the safety and reliability of the FTSST when used with elderly patients following intensive care unit (ICU) discharge, however, the validity of FTSST in this population has yet to be investigated.

Our hypothesis is that the FTSST has the potential to be a valid, reliable, straightforward, and low-cost tool for identifying muscle power and force, walking capacity, fall risk, and functional status following critical disease in younger as well as older adults. Possibly, the FTSST could be used to predict short- and long-term hospital stays when conducted at ICU discharge. Thus, in this study, we sought to: (a) establish the applicability, safety, and reliability of FTSST with patients at ICU discharge and (b) determine the concurrent and predictive validity of the FTSST when used in this way.

## Methods

### Participants

This was a single-center prospective observational study with 142 patients, conducted between June 2017 and May 2018 in a general ICU at Teresa Lisieux Hospital, in Salvador, Brazil. Ethical approval was obtained from the Human Research Ethics Committee of the Federal University of Rio de Janeiro, Brazil, under protocol no. 2,024,986, and all participants provided written informed consent to participate. The study followed the COSMIN Checklist standards to access reliability and validity by using the Classical Test Theory.

### Screening and eligibility

Were included adults of both sexes who were considered functionally independent before hospitalization. We determined functional independence by a score of at least 126 on the Functional Independence Measure (FIM [23];). Additionally, all participants were able to sit-to-stand from a chair five times without any physical assistance at ICU discharge, showed clinical and haemodynamic stability, and received physician approval to walk freely. We excluded individuals with: (a) any substantial pain that might affect participation, (b) cognitive impairment that might interfere with their understanding of test instructions, and/or (c) reduced strength of lower limb muscles (MRC < 5) and (d) other clinical conditions that precluded participation in this test. Members of the clinical staff were instructed to discontinue the test if patients presented dizziness; chest pain; sweating; palpitations; nausea, presyncope, dyspnea; falling; pain or muscle fatigue; peripheral oxygen saturation (SpO_2_) decrease below 92% with or without O_2_ support, respiratory rate (RR) > 22 incursions per minute (ipm), heart rate > 120 beats per minute (BPM), systolic blood pressure (SBP) > 180 mmHg and/or diastolic blood pressure > 100 mmHg, and subjective perception of exertion > 13 evaluated by the Borg Scale of perceived exertion [24].

### Procedure

Senior physiotherapists applied the tests when patients were discharged from the ICU and taken to the hospital ward. Firstly, to determine interrater reliability, two physiotherapists independently applied FTSST on the same day. After an interval of at least 5 minutes since the second evaluation, a third assessment was performed by one of the assessors allowing the calculation of intra-examiner reliability.

As the FTSST reproduces the act of sitting and standing for five repetitions as rapidly as possible [7], test performance was based on how long it took participants to complete these tasks. The shorter times required were intended to better reflect the participant’s functional capacity. Participants began the FTSST by sitting on an armless chair with 43 cm (cm) of seat height [25]. Each participant was instructed to cross their arms over their chest and sit with their back against the upright backrest of the chair. The rater then demonstrated the correct technique for performing the test, including coming to a full stand, defined as an upright trunk with hips and knees extended. Timing began when the rater spoke the word “go” and stopped when the participant’s buttocks reached the seat following the fifth stand [21]. The raters required the patients to stand and sit five times “as quickly as possible” without physical assistance. We did not use words of encouragement or body language to suggest participants to accelerate their performance; rather, we allowed participants to choose their own exercise intensity. If the participants stopped during the test to rest, the raters would say, “You can stay seated if you would like and then continue standing whenever you feel able,” and would not stop the timer. If the patient stopped before completing five repetitions and refused to continue, we registered the reason for him having stopped prematurely and excluded the participant’s score from further analysis.

We used a Dixtal® multi-parameter monitor (DX 2020, Philips, Brazil) to record vital signs data (heart rate, respiratory rate, peripheral oxygen saturation, systolic blood pressure, diastolic blood pressure and double product), and we gave participants a printed version of the traditional Borg Scale [26] for them to rate their perceived exertion. We measured the participant’s vital signs at the beginning and at the end of the test, and we recorded the frequency and type of any adverse events using a checklist including dizziness, fall, dyspnea, chest pain, and musculoskeletal pain. Concurrent validity was assessed using handgrip strength (which is associated with total muscle strength [27];), ICU length of stay, duration of invasive ventilation, the clinical severity score - Simplified Acute Physiology Score 3 (SAPS3 [28];), and age variables. We investigated predictive validity through correlations of the FTSST with such other measures as hospital length of stay and the following domains of the Functional Independence Measure (FIM) – mobility and transfers, locomotion, cognition domains and the change in global FIM scores from pre-hospital to hospital discharge.

## Statistical analysis

We conducted statistical analyses with the Statistical Package for the Social Sciences software version 22.0 (IBM® SPSS®, v. 22.0, Armonk, NY, USA) and Microsoft Excel 2011 (Microsoft Corporation, Redmond, WA, USA). Applicability was determined by calculating the percentage of patients who could perform the FTSST (after the exclusion of patients who deceased, and those who refused to participate). We used the Kolmogorov-Smirnov test to assess the normality of quantitative parameters. We applied the Student’s *t*-test for paired samples to test whether haemodynamic and respiratory variables responses were statistically different between pre- and post-evaluation. Spearman correlation coefficients were calculated between the FTSST and other criterion measures to determine concurrent and predictive validity. We considered a correlation coefficient of .00–0.30 to be very weak, 0.31–0.50 weak, 0.51–0.70 moderate, 0.71–90 strong and 0.91–1.00 very strong as proposed by Mukaka [29].

We determined test-retest and inter-examiner reliability by using the intra-class correlation coefficients (ICCs) and Bland-Altman plots. ICCs were calculated using a two-way random effects consistency model. ICC values less than 0.5 were indicative of poor reliability, values between 0.5 and 0.75 indicated moderate reliability, values between 0.75 and 0.9 indicated good reliability, and values greater than 0.90 indicated excellent reliability as reported by Koo and Li [30]. Standard error of measurement (SEM) was calculated to determine absolute reliability. Predictive validity used the Receiver Operating Characteristic analysis and Youden’s method to determine the best threshold and predictive power of FTSST related to ICU readmission. The level of significance was set at 0.05 for all statistical analysis.

## Results

**Fig. 1:**
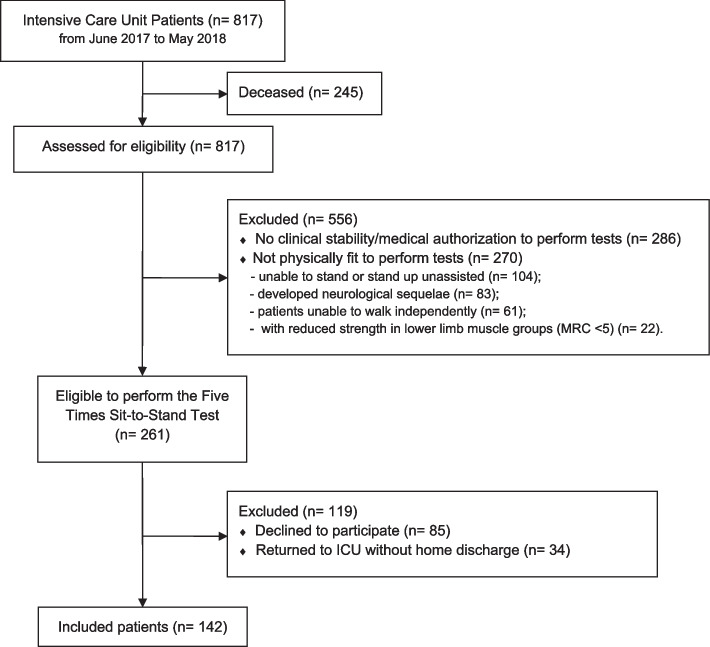
Flow chart of patient selection process

Figure 1 shows a flow chart of participant entry into the study, noting that 261 patients (24.6% of all potential candidates) initially met the inclusion criteria to participate, however, after the exclusion of patients who deceased, and those who refused to participate we observed that only 30% of ICU survivors could perform the FTSST.

Clinical and demographic data of the study population are shown in Table 1. These evaluated patients were 52.8% male, had a median age of 51 years (IQR = 43–64), and 25.4% were over 60 years of age. Regarding their admission diagnosis, 85.2% were admitted for varied clinical treatments – 28.9% for heart conditions and 14.8% for surgical treatment (4.2% for cardiothoracic surgery, 10.6% for neurosurgery). Additionally, we observed that 21.8% used invasive ventilatory support. Patients presented a median SAPS3 of 40 (IQR = 36–57) at admission. The median length of ICU stay was 3 days (IQR = 2–5), with 30.9% of participants remaining for more than 3 days.

**Table 1:** Clinical and demographic characteristics

Characteristics	***N*** = 142
**Age, (years)**	51 [43–64]
**Gender**	
Male	75 (52.8)
Female	67 (47.2)
**Height, (m)**	1.68 [1.60–1.73]
**Weight, (kg)**	74.5 [45–111]
**BMI, (kg/m**^**2**^**)**	25.6 [22.8–29.2]
Normal weight (%)	64 (45.1)
Overweight (%)	78 (54.9)
**Admission diagnosis**	
Neurologic	38 (26.8)
Cardiac	41 (28.9)
Respiratory	28 (19.7)
Nephrological	6 (4.2)
GIT	6 (4.2)
Neurosurgery	15 (10.6)
Cardiac Surgery	6 (4.2)
Obstetrics	2 (1.4)
**Simplified acute physiologic score (SAPS3)**	40 [36–57]
**Use of invasive ventilatory support**	31 (21.8)
**Sepsis**	29 (20.4)
**ICU length of stay, (days)**	3 [2–5]
**Hospital length of stay, (days)**	6 [4–9]
**Handgrip strength, (kgf)**	27.5 [18–35]
**FTSST performance (s) at ICU discharge**	13 [11–15]
**FIM at hospital discharge**	
Self-care	42 [40–42]
Sphincter control	14 [14–14]
Mobility and transfers	21 [18–21]
Locomotion	14 [12–14]
Communication	14 [13–14]
Cognition	21 [19–21]
Global Score	126 [118–126]

Values expressed in median (IQR) or n (%). *BMI* Body mass index, *GIT* Gastrointestinal tract, *FTSST* Five times sit-to-stand test, *FIM* Functional Independence Measure

### Safety assessment

We performed 284 measurements with the FTSTS and only 10 (7%) of the 142 assessed patients presented adverse events during the first time of the test administration, which led to no second FTSST administration. More specifically, five (50%) presented dyspnea, two (20%) experienced muscle pain, two (20%) experienced muscle fatigue and one (10%) experienced chest pain. The test scores of these 10 patients were not considered in the final analysis. When comparing the participants’ physiological data for heart rate, respiratory rate, peripheral oxygen saturation, systolic blood pressure, diastolic blood pressure, and double product, before and immediately after the test, the changes observed were compatible with a normal exercise response without any associated adverse event (Table 2).

**Table 2:** Hemodynamic and respiratory variables pre- and post-Five-Times Sit-to-Stand Test (FTSST)

Variables	Pretest	Post-test	*p*-value
HR (bpm)	77 (14.2)	89 (17.5)	< 0.001
SBP (mmHg)	120 (14.3)	139 (17.5)	< 0.001
DBP (mmHg)	74 (16.4)	83 (12.6)	< 0.001
DP (mmHg.bpm)	9.240 (12.5)	12.4 (13.4)	< 0.001
SPO_2_ (%)	96 (1.9)	98 (1.8)	< 0.001
RR (ipm)	15 (1.5)	18 (2.0)	< 0.001

Values are expressed in mean (±SD). *HR* Heart rate, *SBP* Systolic blood pressure, *DBP* Diastolic blood pressure, *DP* Double product, *BORG (PES)* Perceived exertion score, *SPO*_*2*_ Peripheral oxygen saturation, *RR* Respiratory rate. T-test for paired samples, (*p* < 0.005)

### Reliability assessment

The reliability data shown in Table 3 illustrate that both inter (ICC = 0.92, CI95% 0.89–0.94) and intra-rater (ICC = 0.95, CI95% 0.93–0.96) reliability for this application of the FTSST with these patients were excellent, according to ICC cut-off values reported by Koo and Li [30].

**Table 3:** Five times sit-to-stand test measurements by different examiners and test-retest by the same examiner in hospitalized patients at ICU discharge

Test	Median	IQR	ICC[95% CI]	SEM[95%CI]
Examiner 1	13 s	11–15 s	0.92[0.89–0.94]	0.01 [0.00–0.01]
Examiner 2	12 s	10–14 s		
**Retest**			0.95[0.93–0.96]	0.01 [0.00–0.01]
Examiner 1	12 s	11–15 s		

^a^*IQR* Interquartile range 95%, *CI* Confidence interval, *SEM* Standard error of the mean

**Fig. 2:**
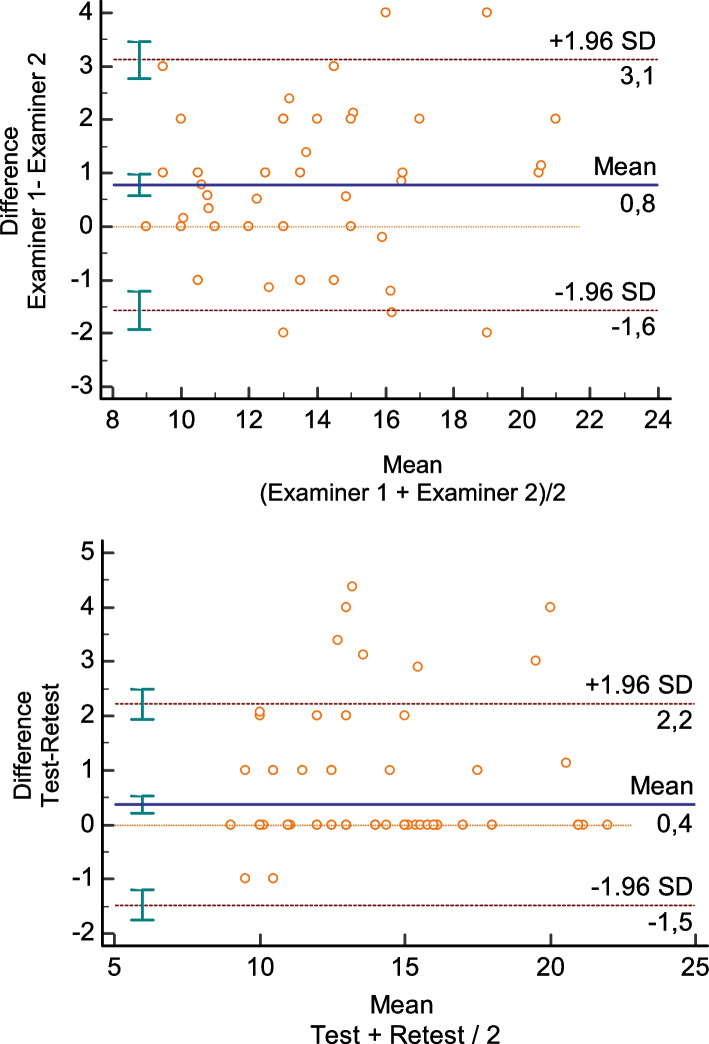
Absolute reliability analysis through the Bland-Altman graphical representation (mean vs. difference) between gait speed (GS) measurements by the same examiner and by different examiners, respectively

The Bland-Altman graphical representation (Fig. 2) showed no systematic error among two trials of the FTSST performed by the same examiner and by different examiners, respectively. The limits of agreement (LOA) were considered acceptable and reflect small differences between paired measurements assessed. Furthermore, a linear regression analysis revealed no proportional bias for inter-rater measurements (*p* = 0.25) and intra-rater measurements (*p* = 0.57).

### Validity

As shown in Table 4, the Five Times Sit-to-Stand Test performance of these critical care survivors presented a moderate correlation with the FIM domains of mobility and transfers (*R*_*s*_ = − 0.67), FIM locomotion (*R*_*s*_ = − 0.66), total FIM score *R*_*s*_ = 0.67), handgrip strength (*R*_*s*_ = − 0.57), self-care and hospital length of stay *(R*_*s*_ = − 0.56) and low correlation with ICU length of stay (*R*_*s*_ = − 0.49). In addition, we found a very weak correlation with FIM sphincter control (*R*_*s*_ = − 0.15), FIM communication (*R*_*s*_ = − 0.18), the SAPS3 clinical severity score (*R*_*s*_ = − 0.20) and duration of ventilatory support (*R*_*s*_ = 0.27).

**Table 4:** Spearman correlation between the five times sit-to-stand test at ICU discharge and clinical and demographic variables

Variable	R_**s**_	***p***-value
Age (years)	−0.11	0.20
Body mass Index (kg/m^2^)	0.07	0.40
SAPS3 score	0.20	< 0.001
ICU length of stay (days)	0.49	< 0.001
MV duration (hours)	0.27	< 0.001
Hospital length of stay (days)	0.56	< 0.001
Handgrip strength (kgf)	−0.57	< 0.001
FIM mobility and transfer domain	−0.67	< 0.001
FIM locomotion domain	−0.66	< 0.001
FIM cognition domain	−0.45	< 0.001
FIM self-care domain	−0.61	< 0.001
FIM sphincter control	−0.15	< 0.03
FIM communication domain	−0.18	< 0.03
FIM global score	−0.67	< 0.001
∆ FIM (previous – discharge)	0.67	< 0.001

*SAPS3* Simplified acute physiologic score 3, *ICU* Intensive care unit, *MV* Mechanical ventilation, *FIM* Functional independence measure

**Fig. 3:**
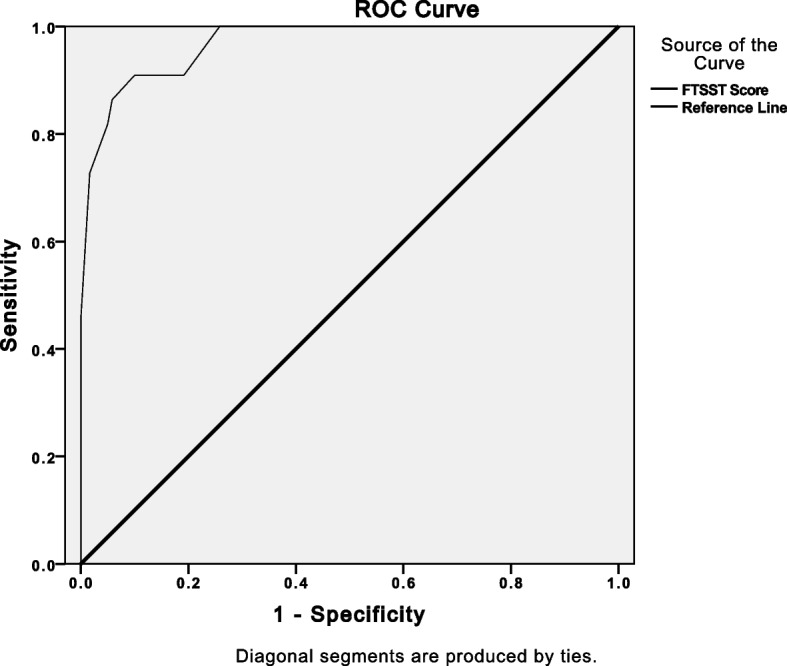
Receiver operating characteristic (ROC) curves demonstrating the ability to predict ICU readmission

A threshold of 23.5 seconds was linked to ICU readmission (AUC 0.94, 95%CI 0.75–0.90, SE 0.16, Sensibility 99%, Specificity 71%). A Receiver operating characteristic curve is provided below (Fig. 3).

## Discussion

Our purpose in this study was to determine the applicability, safety, validity, and test-retest and inter-rater reliability of the FTSST when used with critical care survivors from a general ICU at the time of ICU discharge.

As seen, only 30% of ICU survivors could perform the FTSST and 7% of patients presented with adverse events during the FTSST. Both inter-and intra-rater (test-retest) reliability were excellent (ICC > 0.95, *p* = 0.001) and higher scores were associated with lower muscle strength, longer hospital stay, and greater functional impairment at hospital discharge in adult survivors of critical diseases. Furthermore, a cutoff of 23.5 seconds was predictive of ICU readmission. We advocate that patients with FTSST scores higher than 23.5 seconds may be exposed to considerable risk of ICU readmission and these values should be used to guide physical recovery in order to mitigate such risk. We suggest that this threshold should be tested in another sample of critical care survivors.

Regarding test applicability, it is important to underline that, despite the relatively low median age of our sample, the clinical use of FTSST had to be restricted only to high-functioning post-ICU patients as Melo et al. [9] already reported.

We found the test to be safe with the remaining participants, as demonstrated by hemodynamic and respiratory variables responses (pre- and post-test) and a low rate (7%) of adverse events. Previous studies [9, 10] that used the FTSST in a hospital setting have also reported an absence of important adverse events.

Regarding test reliability, results revealed excellent test-retest and inter-rater reliability, with low percentages of error measurement, as shown by reliability methods, including ICCs and visual inspection of the Altman-Bland plot. This finding is in accordance with other studies that have investigated the test-retest and inter-rater reliability of the FTSST [9, 31–34]. This high inter-rater and test-retest reliability was deemed possible because of the straightforward test instructions, the researchers’ experience, and the objective nature of the test assessment.

Our study is the first to investigate the concurrent and predictive validity of the FTSST in patients at ICU discharge. The study of its clinical applicability through the safety, validity, and reliability of measurements is essential to allow a more accurate and earlier analysis of the functional status of critical care survivors. Our results suggest that the FTSST performance at ICU discharge is a clinically valid measure for determining muscle performance and functional status at hospital discharge. It was clear that patients with higher FTSST scores tended to show less peripheral muscle strength as evidenced by lower handgrip strength and lower functional scores in many FIM domains (mobility and transfers, locomotion, cognition, self-care and global score) at hospital discharge. These findings are important as the early performance of functional status evaluation may identify patients who are at higher risk for functional decline after hospital discharge and possible increased post-discharge mortality [35].

A recent study developed by Mayer et al. [10] has shown that the FTSST is highly associated with muscle strength in critical survivors and therefore could be used to reflect muscle dysfunction and physical disability at ICU discharge. Since instruments to assess muscle function generally are not available at bedside, the FTSST seems to be a practical and reproducible option to record muscle strength in hospitalized patients. Moreover, it is known that the FTSST has already been associated with reduced strength in the lower limbs [11], balance control [13], risk of falls [12] in older subjects, and risk of fall and functional recovery management in a hospital setting.

Interestingly, the FTSST performance in our study showed a moderate correlation with hospital length of stay and a moderate correlation with ICU length of stay and duration of ventilatory support. In other words, survivors of critical illness with lower scores at ICU discharge apparently tended to stay longer in the hospital, possibly due to greater physical fragility. Ventilatory support and ICU length of stay are suggested as potential contributors of functional deterioration as stated in previous studies [36–39].

Even though previous authors have found a strong association between clinical severity scores at admission and physical impairment [40], this association was not strong in our study, probably due to the severity profile of the included patients.

The present study is the first investigation of the FTSST applicability in a sample of survivors of critical disease at ICU discharge other than older subjects. The study findings suggested that the FTSST appears to be a safe, valid, reliable, and straightforward resource with the potential to contribute to the evaluation of the functional status performance of critical care survivors. The application of FTSST in this critical setting may also be useful to identify patients at risk of low functional recovery and falls during the hospital stay and after hospital discharge so as to guide the rehabilitation treatment.

Further studies are required to investigate the relationship between FTSST scores and short- and long-term outcomes, including hospital readmission rate and mortality. The main limitations of this study are the use of a convenience sample and a single center recruitment experience, as well as the relatively low median age of the patients included.

## Conclusion

Our results suggest that the FTSST may be applicable only to high-functioning critical care survivors. In this specific population, FTSST is a safe, easy to perform, valid, and reliable measure that can be applied to support fall risk and functional recovery management.

## Data Availability

The dataset analysed during the current study is available from the correspondent author on reasonable request.
