# Correction: The five times sit-to-stand test: safety, validity and reliability with critical care survivors’s at ICU discharge

**DOI:** 10.1186/s40945-023-00161-w

**Published:** 2023-03-07

**Authors:** Thiago Araújo de Melo, Fernando Silva Guimarães, José Roberto Lapa e Silva

**Affiliations:** 1 School of Health Sciences – Brazil, Universidade Federal do Rio de Janeiro, Rua das Patativas, 449, Imbuí, Salvador, Bahia Brazil; 2 Physical Therapy Department, Federal University of Rio de Janeiro, Rua prof. Rodolpho Paulo Rocco, 255, oitavo andar, sala 3 - Cidade Universitária da Universidade Federal do Rio de Janeiro, Rio de Janeiro, RJ Brazil; 3 School of Medicine – Federal University of Rio de Janeiro, Rua Ferreira Pontes, 264/103, Andaraí, Rio de Janeiro, RJ Brazil

**Correction: Arch Physiother 13, 2 (2022)**

**https://doi.org/10.1186/s40945-022-00156-z**

Following the publication of the original article [1] it was brought to our attention that the letters A. B. C. that had been used to number the three co-authors, were unfortunately captured as author initials.

The correct author names are: Thiago Araújo de Melo, Fernando Silva Guimarães and José Roberto Lapa e Silva.

These are shown in the author list of this Correction as well as the original article, which has already been revised.
